# Testosterone level and the effect of levodopa and agonists in early Parkinson disease: results from the INSPECT cohort

**DOI:** 10.1186/2054-7072-1-8

**Published:** 2014-11-26

**Authors:** Michael S Okun, Samuel S Wu, Dana Jennings, Kenneth Marek, Ramon L Rodriguez, Hubert H Fernandez

**Affiliations:** Departments of Neurology, Neurosurgery and Psychiatry, McKnight Brain Institute, University of Florida, 100 S Newell Dr Rm-L3-101, Gainesville, 32611 FL USA

**Keywords:** Testosterone, Parkinson’s disease, Levodopa, Agonist, Medications, LH

## Abstract

**Background:**

To determine if testosterone levels are influenced by dopaminergic therapy in Parkinson disease (PD) patients. Testosterone level has been reported to be low in patients with PD and other neurodegenerative diseases. In this study, we sought to determine whether dopaminergic therapy (i.e. levodopa and dopamine agonist) influenced testosterone levels. We used a cohort of consecutive male patients from the INSPECT trial--a multi-center, prospective, study that primarily investigated the effects of short-term treatment with pramipexole or levodopa on [^123^I] B-CIT SPECT imaging in early PD.

**Methods:**

Testosterone levels were drawn on consenting male subjects with early PD who enrolled in the INSPECT trial at three study visits (baseline, 12 weeks post-treatment, and 8–12 weeks post-washout). Subjects were randomized to: no treatment, pramipexole (up to 3 mg) or levodopa (up to 600 mg). Testosterone levels were obtained twice (prior to 10 AM) and averaged for each of three study visits.

**Results:**

Thirty two male patients participated in this sub-study and there were no significant differences in disease characteristics in the 3 groups at baseline. Twenty-nine patients completed the follow-up visits and were suitable for analysis. There were statistically significant differences in the change in free testosterone level, increased in both the levodopa group and pramipexole group but decreased in the untreated group at 12-weeks post-treatment. There were no significant differences in the changes of UPDRS total or motor scores, although there was a strong trend toward improvement in motor scores. The testosterone level persisted in its increase only in the pramipexole group at the end of the washout period.

**Conclusion:**

These preliminary data support the premise that dopaminergic medications do not reduce testosterone levels in early PD patients.

**Electronic supplementary material:**

The online version of this article (doi:10.1186/2054-7072-1-8) contains supplementary material, which is available to authorized users.

## Background

There has been mounting evidence suggesting that inappropriately low plasma testosterone levels commonly exist in male patients with Parkinson disease (PD) [1–6]. The relevance and underlying cause of this endocrine disturbance remain unknown. Additionally, replacement of testosterone has not to date been proven to significantly improve the motor and non-motor symptoms of these patients, though there have been anecdotal successes [4–7]. Two main hypotheses have been proposed to explain the low testosterone levels: the levels are reduced by dopaminergic medications, or the testosterone level is a surrogate marker for pathology known to occur in the hypothalamus and in other relevant regions of the PD brain [4]. We sought to examine the former hypothesis that dopaminergics may lower testosterone. In this study we utilized the INSPECT cohort of early PD subjects.

## Methods

**Figure 1:**
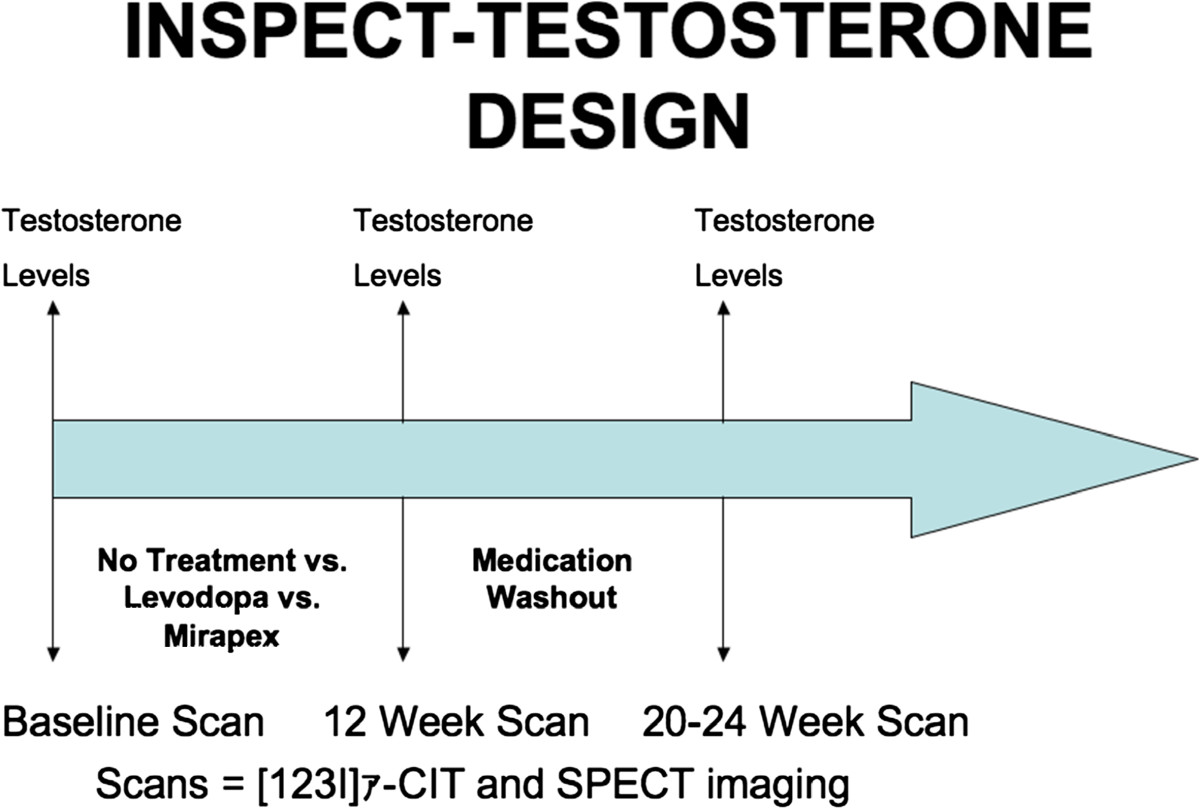
**Study design.**

Consecutive male PD patients (who fulfilled the UK Parkinson’s Disease Brain Bank Criteria [8]) enrolled in the INSPECT study had plasma free and total testosterone levels drawn. Patients had two testosterone levels (free and total) drawn prior to 10 AM during each study visit as described in a previous study [4]. The levels for each subject at each study interval were averaged. Three scans- [123I] -CIT and SPECT imaging were performed at baseline, 12 weeks following randomization to treatment group (levodopa [up to 600 mg/day], pramipexole [up to 3 mg/day], or no treatment), and 8–12 weeks following medication washout (see Figure 1). Baseline levels and descriptive clinical variables were recorded, and changes from baseline to 12 weeks post-treatment, and changes from baseline to 8–12 weeks post washout were compared between the three groups using analysis of variance (ANOVA).The University of Florida Institutional Review Board (IRB) through the parent INSPECT study approved the investigation, and each participant provided informed consent.

## Results

**Figure 2:**
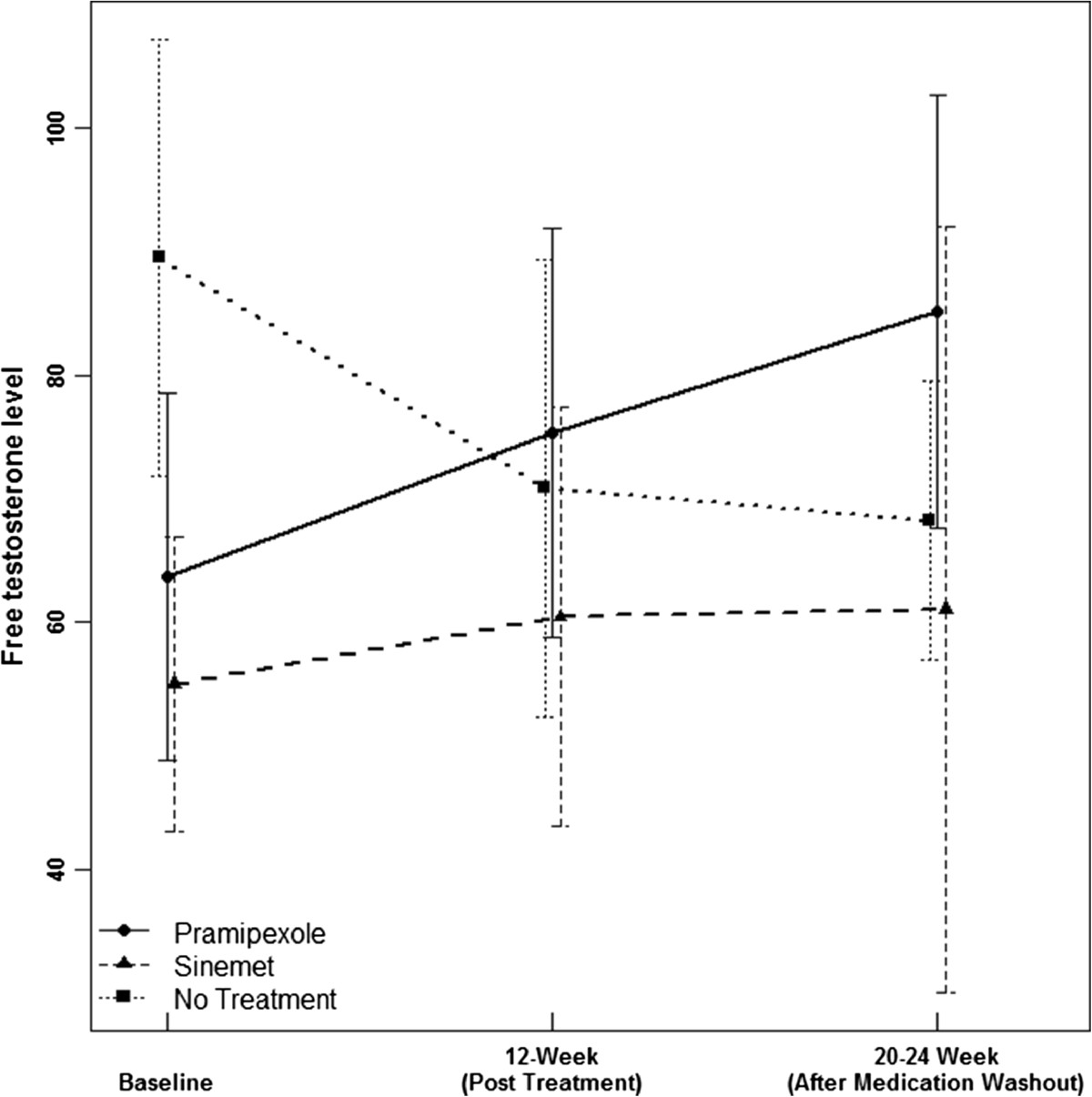
**Mean and 95% confidence interval of the free testosterone level by time and group.**

Thirty-two consecutive male patients enrolled in the study and no differences in disease characteristics between groups were seen at baseline (Table 1). Twenty nine patients completed the follow-up visits and were suitable for analysis. Figure 2 shows the mean and 95% confidence interval of the free testosterone level by time and group. The free testosterone level increased in both the levodopa group (change of 5.41 ± 16.18) and pramipexole group (8.95 ± 13.15), but decreased in the untreated group (−13.92 ± 23.12) from baseline to 12-week post-treatment. The between group difference reached a statistical significance of 0.019 with the use of an ANOVA test on the changes. There were no significant changes in UPDRS total or motor scores although there was a trend toward improvement in motor scores in both treatment groups compared to placebo. The increase in testosterone level persisted only the pramipexole group post-washout (see Tables 2 and 3).

**Table 1:** **Baseline characteristics of subjects in the study**

Group	N	Age		PD duration		UPDRS M		UPDRS T		Free T		Total T	
		Mean	SD	Mean	SD	Mean	SD	Mean	SD	Mean	SD	Mean	SD
**Pramipexole**	**12**	68.83	13.13	2.44	1.67	21.92	5.62	30.75	8.25	63.69	25.24	386.32	151.73
**Levodopa**	**9**	70.33	9.31	2.11	1.45	17.44	7.09	25.22	10.51	55.03	18.18	361.06	146.71
**NoTreatment**	**11**	60.36	6.55	2.1	1.6	17.91	8.23	26.09	11.41	89.54	29.88	449.55	134.92

Legend: UPDRS M- Unified Parkinson Disease Rating Scale Motor Subsection, UPDRS T- Unified Parkinson Disease Rating Scale total score. Free T - free testosterone level; Total T - total testosterone level.

**Table 2:** **Summary of changes from baseline to 12 weeks post treatment**

Group	N	Total T		UPDRS M		UPDRS T	
		Mean	SD	Mean	SD	Mean	SD
**Pramipexole**	**10**	34.80	87.33	−4.27	6.15	−5.91	7.34
**Sinemet**	**9**	−34.17	59.79	−6.11	6.77	−9.11	10.62
**NoTreatment**	**10**	21.80	74.71	0.90	6.64	−0.50	8.87
		p = 0.12		p = 0.06		p = 0.12	

Legend: Total T - total testosterone level. UPDRS M- Unified Parkinson Disease Rating Scale Motor Subsection; UPDRS T- Unified Parkinson Disease Rating Scale total score. Free testosterone measured in pg/ml, and total testosterone in ng/dl.

**Table 3:** **Summary of changes from baseline to the end of washout period**

Group	N	Total T		UPDRS M		UPDRS T	
		Mean	SD	Mean	SD	Mean	SD
**Pramipexole**	**7**	103.79	49.47	0.89	5.62	1.56	5.29
**Sinemet**	**7**	−13.21	40.27	1.86	6.54	1.71	7.93
**NoTreatment**	**9**	100.78	104.58	0.78	8.63	−0.22	11.39
		p = 0.01		p = 0.95		p = 0.88	

Legend: Total T - total testosterone level. UPDRS M- Unified Parkinson Disease Rating Scale Motor Subsection; UPDRS T- Unified Parkinson Disease Rating Scale total score. Free testosterone measured in pg/ml, and total testosterone in ng/dl.

## Discussion and conclusions

These results suggest that neither levodopa nor pramipexole decrease testosterone level in early PD. The observation that the untreated group experienced further lowering of free testosterone levels lends support to the hypothesis that testosterone decline in PD may be a result of disease-specific factors, and that the decline is less likely iatrogenically induced by dopaminergic medications. It is not entirely clear why the increase in free testosterone levels persisted in the dopamine agonist group post-washout. Dopamine agonists have been used in the treatment of prolactinoma, because dopamine is a natural inhibitor of prolactin [9, 10]. Prolactin lowers leutenizing hormone (LH) which in turn lowers testosterone level [11–16]. Thus, dopamine agonists may theoretically increase testosterone levels by inhibiting prolactin. This point will need further clarification.

Sinhamahapatra and Kirschner in 1971 published a detailed analysis of the effect of levodopa on testosterone level [17]. They sought in their study to answer the question as to whether levodopa stimulated LH production and Leydig cell activity. They utilized an electron capture gas liquid chromatographor, an older technique now considered less accurate when compared to more modern techniques for measuring testosterone. Seven PD men staged using an older system devised by Leon were included in the analysis . Very high doses of levodopa were used (2–6 grams/day). There were other methodological limitations including baseline normal levels in all patients enrolled (>325 ng/ml), and 2/7 PD patients who did not improve on levodopa. One interesting aspect of their study was the calculation of testosterone production and metabolism, as well as the measurement of LH. The study concluded that levodopa did not have an impact on plasma testosterone or LH [17]. Despite methodological limitations this data is supportive of our findings.

We suspect based on our results that the finding of low testosterone in PD patients is indicative of intrinsic PD pathology. It is well known that Lewy Body pathology is present in PD patients at post-mortem examination, and lesions includes hypothalamic involvement [18–22]. Braak has shown that this hypothalamic pathology may be present relatively early in the course of PD [18–21]. We propose this as a plausible explanation for the low testosterone levels.

There were several limitations in this study that should be addressed in future investigations. These limitations included a small sample size, the lack of non-PD controls, the use of early PD patients, differences between free and total testosterone levels, and observed changes in testosterone level were small and not likely clinically relevant. There were changes seen between free testosterone and total testosterone and this highlighted difficulties in laboratory measurements. Most experts use a free or bioavailable testosterone level as the gold standard rather than utilizing both a free and total testosterone level [23–25]. Despite these limitations we conclude as did Sinhamahapatra, that dopaminergics are probably not the cause of low testosterone in PD. Clinicians should not assume that low testosterone levels are an effect of PD medications. There is currently no evidence that checking a testosterone level prior to dopaminergic therapy will be clinically useful. We suggest that future research on this topic should focus on disease related factors as the potential culprits in the low testosterone PD story, however a larger study of the effects of medication can confirm our results.

## Authors’ original submitted files for images

Below are the links to the authors’ original submitted files for images. Authors’ original file for figure 1 Authors’ original file for figure 2
