# Supplementary figures and images for: Testosterone level and the effect of levodopa and agonists in early Parkinson disease: results from the INSPECT cohort
Source: J Clin Mov Disord. 2014 Nov 26;1:8. doi: 10.1186/2054-7072-1-8 (PMC4711001; doi:10.1186/2054-7072-1-8)

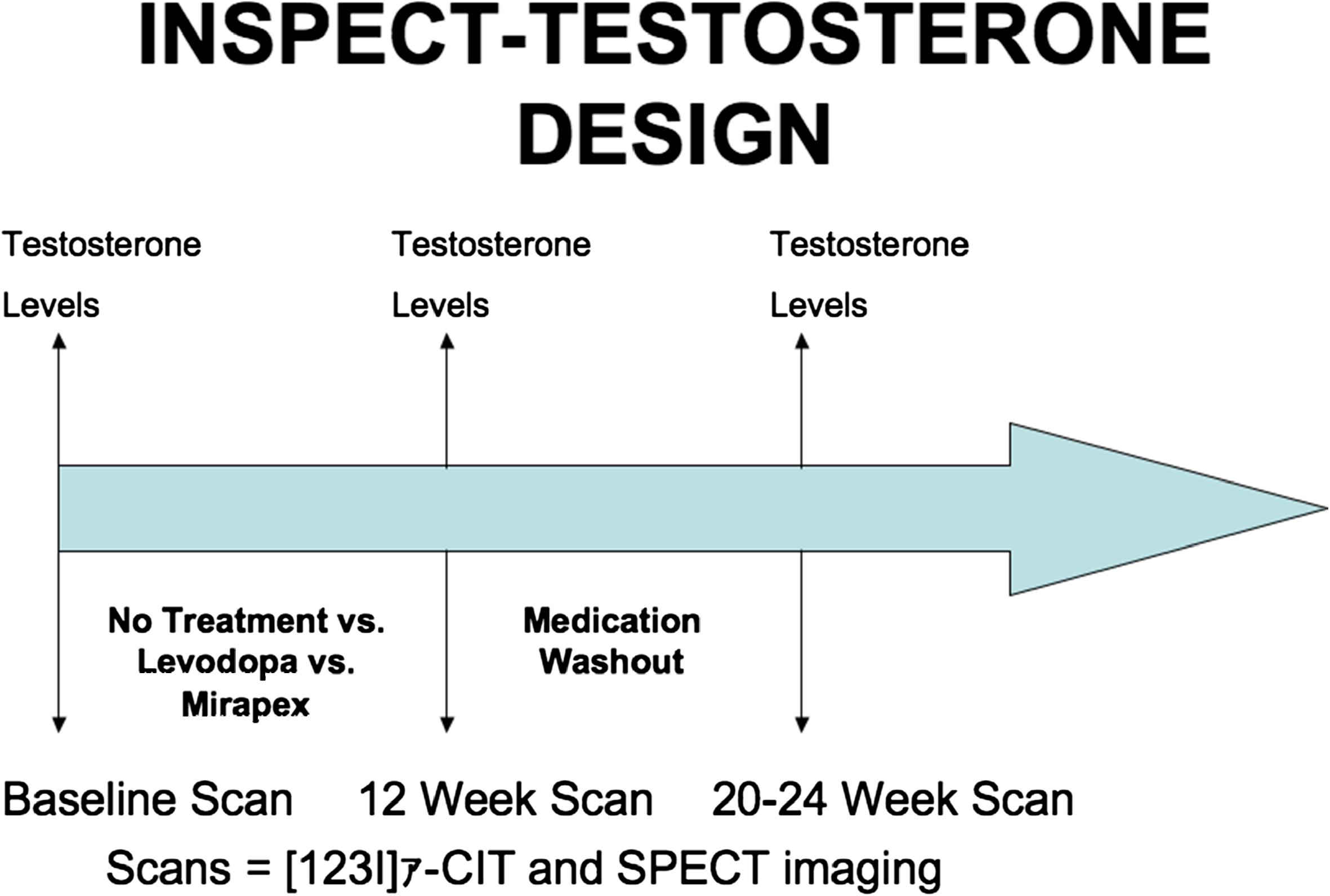

Supplement: Supplementary file 1 — Authors’ original file for figure 1 [file 40734_2014_23_MOESM1_ESM.tiff]

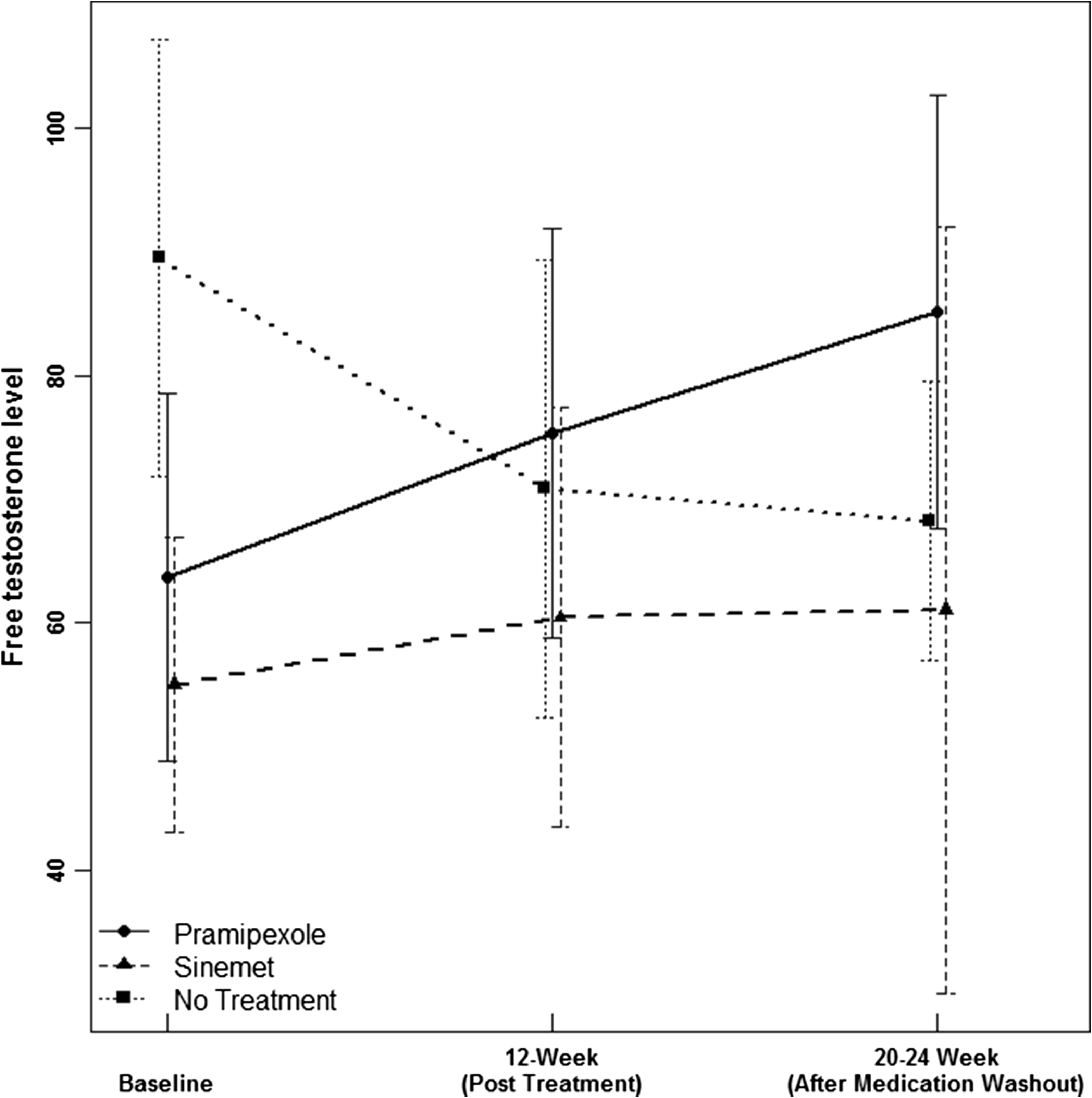

Supplement: Supplementary file 2 — Authors’ original file for figure 2 [file 40734_2014_23_MOESM2_ESM.tiff]
